# ADHD candidate gene (DRD4 exon III) affects inhibitory control in a healthy sample

**DOI:** 10.1186/1471-2202-10-150

**Published:** 2009-12-20

**Authors:** Ulrike M Krämer, Nuria Rojo, Rebecca Schüle, Toni Cunillera, Ludger Schöls, Josep Marco-Pallarés, David Cucurell, Estela Camara, Antoni Rodriguez-Fornells, Thomas F Münte

**Affiliations:** 1 Dept. of Neuropsychology, Otto-von-Guericke-University, Magdeburg, Germany; 2 Helen Wills Neuroscience Institute, University of California, Berkeley, USA; 3 Dept. of Physiological Sciences II, University of Barcelona, Barcelona, Spain; 4 Hertie-Institute for Clinical Brain Research, University of Tübingen, Tübingen, Germany; 5 Dept. Psicologia Bàsica, University of Barcelona, Barcelona, Spain; 6 Institució Catalana de Recerca i Estudis Avançats (ICREA), Barcelona, Spain; 7 Center for Behavioral Brain Sciences, Magdeburg, Germany

## Abstract

**Background:**

Dopamine is believed to be a key neurotransmitter in the development of attention-deficit/hyperactivity disorder (ADHD). Several recent studies point to an association of the dopamine D4 receptor (DRD4) gene and this condition. More specifically, the 7 repeat variant of a variable number of tandem repeats (VNTR) polymorphism in exon III of this gene is suggested to bear a higher risk for ADHD. In the present study, we investigated the role of this polymorphism in the modulation of neurophysiological correlates of response inhibition (Go/Nogo task) in a healthy, high-functioning sample.

**Results:**

Homozygous 7 repeat carriers showed a tendency for more accurate behavior in the Go/Nogo task compared to homozygous 4 repeat carriers. Moreover, 7 repeat carriers presented an increased nogo-related theta band response together with a reduced go-related beta decrease.

**Conclusions:**

These data point to improved cognitive functions and prefrontal control in the 7 repeat carriers, probably due to the D4 receptor's modulatory role in prefrontal areas. The results are discussed with respect to previous behavioral data on this polymorphism and animal studies on the impact of the D4 receptor on cognitive functions.

## Background

Considerable evidence exists for an association of the dopamine D4 receptor (DRD4) gene located on chromosome 11p15.5 and attention-deficit/hyperactivity disorder (ADHD) [1-3]. In particular, a specific allele (7-repeat) of a variable number of tandem repeat (VNTR) polymorphism in the coding region of this gene has been suggested to be a risk factor for the development of ADHD. Although there exist a number of association studies [4-6] and a few studies on neuropsychological correlates of this polymorphism in ADHD [2,7,8], evidence regarding the underlying neural mechanisms mediating this association remains scarce [9,10]. However, ADHD is known to imply changes in a range of neurophysiological markers of prefrontal functions such as performance monitoring and inhibitory control [11-13]. Moreover, altered prefrontal functions in ADHD are often related to the assumed underlying dopaminergic dysfunction in those patients [1,3]. The question thus arises whether prefrontal functions are modulated also by the DRD4 VNTR polymorphism. The present study aimed at investigating possible behavioral differences related to the 7-repeat allele as well as its impact on neurophysiological correlates of prefrontal functions in a healthy, high-functioning sample.

The D4 receptor belongs to the D2-like dopamine receptor class and has received special interest in the past, because the atypical neuroleptic clozapine binds with high affinity and specificity to this receptor [14]. The D4 receptor is particularly abundant in prefrontal regions (PFC; including ACC), but expressed also in regions belonging to the limbic system such as the amygdala and hippocampus [15,16]. Regarding its functional role, an inhibitory influence of D4 receptor activation on GABAergic and glutamatergic functions in the PFC has been demonstrated, possibly underlying the beneficial effects of neuroleptics such as clozapine [17-19].

Recently, a number of different polymorphisms both in the promotor and the coding region of the D4 receptor gene have been studied regarding their physiological and behavioral relevance [9,20-23]. The VNTR investigated in the present study is an extensive polymorphic 48bp sequence in exon III that is coding for the third intracellular loop in the D4 receptor [16,24,25]. The 7-repeat variant has been shown to be half as potent in its ability to inhibit cyclic adenosine monophosphate (cAMP) formation compared to the 2- or 4-repeat variants [26]. Importantly, there are by now several meta-analyses providing evidence for a small, but robust association between the 7-repeat variant and ADHD [5,6,27], whereas studies regarding a link of this polymorphism and the personality trait novelty seeking remained inconclusive [28,29].

In the last years, this gene-ADHD association has led to several studies investigating possible neuropsychological effects of the DRD4 VNTR, mainly in ADHD children [2,7,8], but also in healthy participants [30]. However, variability in the included samples regarding participants' disease severity as well as differences in the applied behavioral paradigms and finally contradictory results render conclusions about the genetic effects difficult [for a review see [31]]. Moreover, the majority of these studies were conducted on heterozygous participants, as the allele frequency of the 7 repeat variant is quite low. Swanson and colleagues [2], for instance, investigated attentional control in a sample of children with the ADHD-Combined type. Contrary to their expectations, they found less accurate and more variable performance in patients without the 7 repeat variant, but not in the patients with at least one 7 repeat allele. The authors (and others observing similar results) hence suggested that the 7 repeat variant might present a subgroup with the behavioral but not cognitive symptoms of ADHD [2,7,32]. However, in a healthy sample, Congdon and co-authors [30] reported reduced inhibitory control in carriers of at least one 7 repeat allele compared to participants without one.

Whereas behavioral data thus do not allow final conclusions about the functional role of this genotype, neurophysiological data (such as event-related potentials, ERPs or task-induced oscillations) might be more sensitive for subtle, genetically caused differences [20,33,34] and can moreover speak to the underlying mechanisms of the gene-ADHD association. Electrophysiological studies on the Go/Nogo-paradigm have focussed in particular on the frontal N2/P3 complex in nogo-trials [35,36], supposedly related to the inhibition and its evaluation, respectively [12,37], but see [38,39]. Few studies have analyzed time-frequency changes related to motor inhibition, pointing to both modulations in the theta and beta frequency bands [40-42]. Whereas increases in the theta band over frontocentral areas are seen in a range of cognitively demanding situations [43-45], changes in beta frequency oscillations have been related both to changes in motor excitability and inhibitory frontal control [41,42,46]. More specifically, event-related desynchronization (ERD) in the beta band is typically seen before onset of movement with a rebound (event-related synchronization, ERS) after the movement. ERD and ERS have been proposed to be related to cortical activation and a cortical resting state, respectively [46-48]. Importantly, ADHD is known to be associated with altered prefrontal functions related to behavioral inhibition and action monitoring, which has also been shown to be reflected in diminished ERP components as the nogo-related N2 or the error-related negativity [11,12,49]. Differences in such markers of prefrontal functions might thus help to clarify the neural processes that are affected by this genetic risk factor.

The aim of the present study was thus to investigate the polymorphism's impact on neurophysiological correlates of inhibitory control and to clarify previous contradictory results in behavioral studies. We performed a Go/Nogo task with participants selected from a larger sample based on their DRD4 alleles to investigate neurophysiological correlates (ERPs and task-induced oscillations) of inhibitory control. We used a hybrid go/nogo choice reaction task that allowed us to parametrically manipulate inhibitory functions (see methods). Deficits in inhibitory functions should especially be detectable under more demanding conditions. Such an approach has been proven useful to detect subtle genetically caused differences [50]. Importantly, we included only participants being homozygous for either the 4 repeat or the 7 repeat variant in the EEG sample. As the 7 repeat allele is rare [51], the vast majority of studies has compared heterozygous participants, rendering conclusions about genetic effects in these participants questionable. We found group differences in both performance and electrophysiological effects, which point to improved cognitive functions and prefrontal control in the 7 repeat carriers, probably due to the D4 receptor's modulatory role in prefrontal areas.

## Results

### Behavioral results

Participants' mean reaction time in go trials was 525 ms and they had on average 95.0% of hits and 16.8% of false alarms in the Nogo trials. Participants responded faster in the easy than in the hard condition, although this effect was more pronounced for the high go probability block (72%), resulting in faster responses for the easy trials in blocks with 72% compared to 50% go probability (Discriminability*Probability: F_1,18 _= 5.87, p = 0.026). The two DRD4 groups did not differ with respect to their reaction times (main effect of DRD4 factor and interactions: p > 0.2; Table 1). Participants were significantly less accurate (difference between percentage of hits and percentage of false alarms) in their behavior in the hard (69.7%) compared to the easy condition (93.2%; Discriminability: F_1,18 _= 125.4, p < 0.001).

**Table 1:** Demographic and behavioral data

	4rep	7rep
Sex (F/M)	10/0	8/2
Age (mean, years)	20.0 (1.4)	22.6 (3.4)

RT (easy - 50% go)	495.6 (92.8)	529.0 (70.1)
RT (hard - 50% go)	538.7 (110.7)	568.7 (69.9)
RT (easy - 72% go)	464.3 (91.8)	506.4 (63.0)
RT (hard - 72% go)	539.0 (87.9)	558.0 (68.8)
Hits/False alarms (easy - 50% go)	98.1/2.32	98.2/3.4
Hits/False alarms (hard - 50% go)	95.0/21.2	94.3/20.5
Hits/False alarms (easy - 72% go)	98.1/8.5	98.3/5.6
Hits/False alarms (hard - 72% go)	84.5/23.8	93.8/23.4

RT refers to mean reaction time of hits in ms in the two discriminability conditions (easy, hard) and 50% or 72% go probability blocks. Hits and false alarms are given in percentage values. Numbers in brackets refer to the standard deviance.

**Figure 1:**
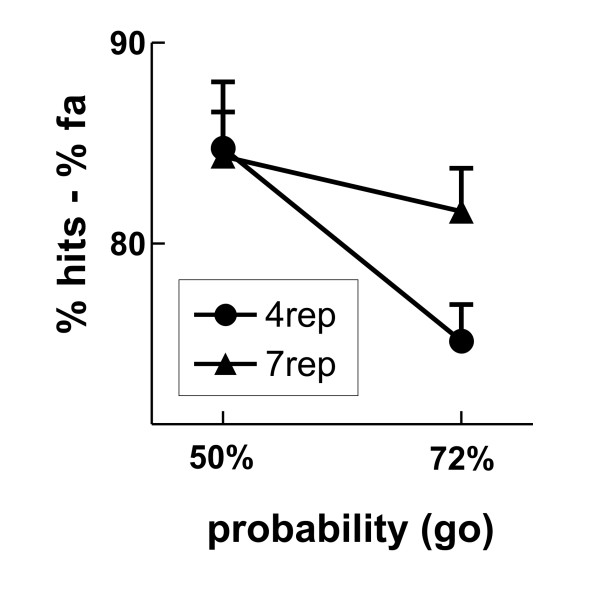
**Behavioral results for the Go/Nogo-task**. Depicted is the average accuracy (% hits -% false alarms) for the two groups (4rep and 7rep), separately for the blocks with 50% and 72% go trials.

We observed group differences in accuracy: The 4rep group showed less accurate behavior in blocks with 72% compared to 50% go trials (75.2 vs. 84.8%; Probability: F_1,9 _= 22.03, p = 0.001), whereas no such block effect was seen in the 7rep group (81.6 vs. 84.3%; Probability: F < 1). However, the interaction yielded only marginal significance (Probability*DRD4: F_1,18 _= 3.82, p = 0.066, partial η^2 ^= 0.18; Figure 1). Similar results were derived when using the signal detection measure a' as dependent variable [52], but the interaction yielded significance here (Probability*DRD4: F_1,18 _= 5.63, p = 0.029, partial η^2 ^= 0.24). As can be seen in Table 1, this group difference was caused mainly by less hits in the 4rep group in the block with 72% compared to 50% go-trials.

### Go/Nogo: ERPs

**Figure 2:**
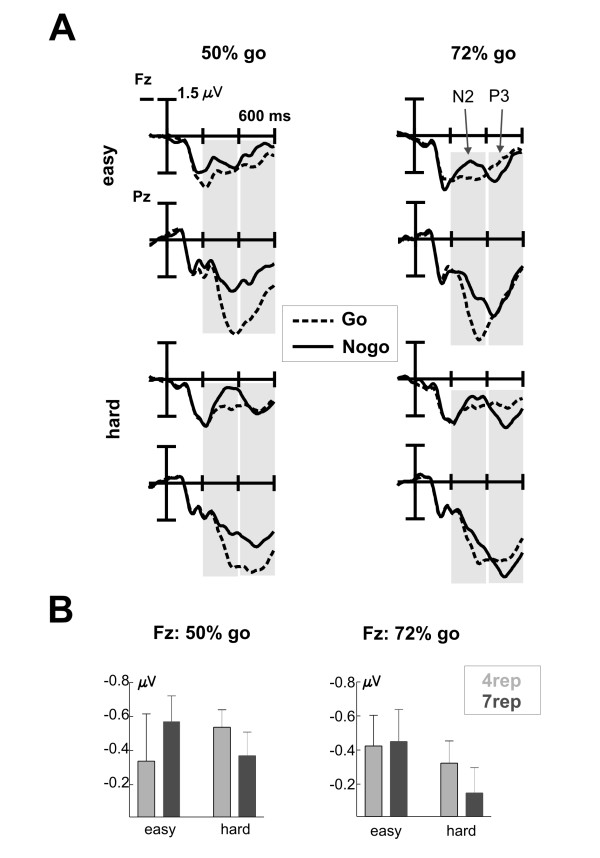
**Event-related potentials (ERPs) in the Go/Nogo task**. **A **Shown are the ERPs in Go (dashed lines) and Nogo trials (solid lines) at frontal (Fz) and posterior (Pz) sites, separately for 50% (left side) and 72% go blocks (right side) and for the easy (upper half) and hard condition (lower half). Marked are the time windows that were analyzed for the N2 and P3a effects. **B **Average difference values (Nogo - Go) with the respective standard error bars at frontal sites (Fz) for the time window 200 - 400 ms (N2). Results are shown separately for the two groups (grey: 4rep and black: 7rep), for 50% (left side) and 72% go blocks (right side) and further separated for the two conditions.

ERPs revealed an enhanced negativity (N2) in nogo compared to go trials around 200 to 400 ms after stimulus onset, followed by a higher nogo-related frontal positivity (nogo-P3; Figure 2A) [37]. At posterior sites, we observed a higher positivity for go compared to nogo trials (P3b), with a maximum around 400 ms. We will report both the effects of the task conditions (discriminability and probability) as well as group differences separately first for the nogo-N2 and then for the nogo-P3.

#### Nogo-N2

The nogo-related N2 increase was clearly significant and differed between the discriminability conditions (easy vs. hard) and the electrode position (Go*Discriminability*Electrode: F_2,36 _= 10.13, p = 0.001), but did not differ between the groups (all interactions involving DRD4 and Go: n.s., all partial η^2 ^< 0.1; Figure 2B). Moreover, contrary to our prediction, the N2 did not increase in 72% go blocks (all interactions involving Probability and Go: n.s.). As can be detected in Figure 2, the conditions differed however in the amplitude of the preceding positivity. We thus additionally computed the amplitude difference at Fz between the N2 peak (mean amplitude in 40 ms window around the peak in nogo-trials, separately assessed for the different conditions) and the preceding positivity (mean amplitude 180-220 ms) and subjected this difference value to a repeated measures ANOVA as outlined above. We found a significant interaction of Go * Probability * Discriminability (F_1,18 _= 8.27, p = 0.01), reflecting a tendency for an increased N2 in the easy condition in 72% compared to 50% go blocks (Go * Probability: F_1,18 _= 3.24, p = 0.089), but a tendency for a decreased N2 in the hard condition in 72% compared to 50% go blocks (Go * Probability: F_1,18 _= 4.31, p = 0.053).

Further evaluation of the interaction Go*Discriminability*Electrode revealed different topographies for the conditions: Whereas in the hard condition the effect was clearly evident at all three midline electrodes (Go: F_1,18 _= 22.58, p < 0.001; Go*Electrode: p > 0.1), the effect did interact with the electrode position in the easy condition (Go*Electrode: F_2,36 _= 8.79, p = 0.002). However, contrary to the typical N2 topography with a frontocentral maximum, the Nogo-related difference was maximal at the posterior electrode (Pz). In fact, as can be clearly asserted from Figure 2A, the frontal N2 effect was overlapping with the posterior P3b effect, i.e. an enhanced positivity for Go compared to Nogo stimuli. This temporal overlap of the two components makes a clear assessment of the nogo-N2 and putative group differences difficult. It might well be that group differences in the nogo-N2 are overshadowed by the larger P3b effect.

#### Nogo-P3

The frontal nogo-P3 effect, which seems to be present in the 72% block, did not reach significance. Although we observed an interaction of Go*Probability*Electrode (F_2,36 _= 14.18, p < 0.001) in the time-range of the nogo-P3 (400 - 600 ms), this was due to differences in the posterior P3b. More specifically, a long-lasting P3b effect was observed in the 50% go block, with a maximum over posterior electrodes (Go*Electrode: F_2,36 _= 12.5, p < 0.001; Figure 2A). Importantly, no main effect or interaction with the factor Go yielded significance in the 72% go blocks (all: p > 0.1). Neither did any interaction involving the group factor reach significance (all F < 1, all partial η^2 ^< 0.1).

### Go/Nogo: Time-frequency results

**Figure 3:**
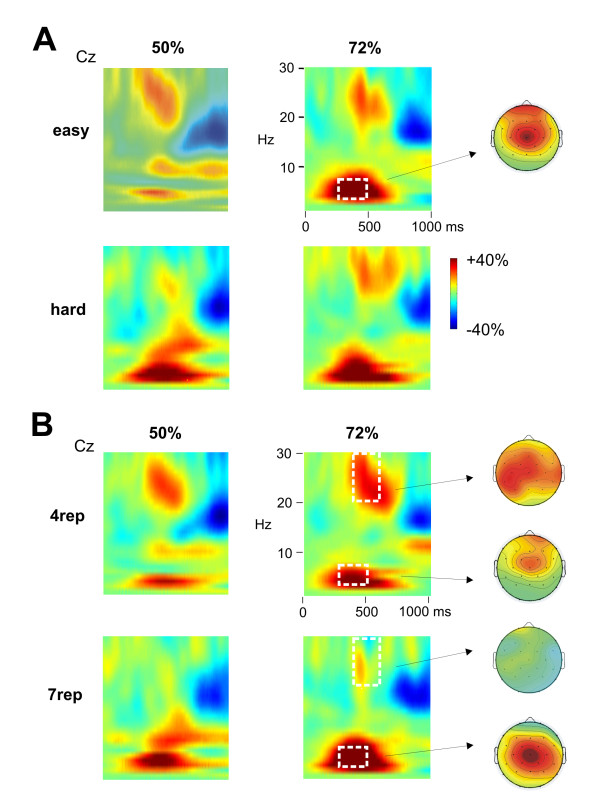
**Time frequency results of the Go/Nogo task**. **A **Differences in the power change relative to the baseline between Nogo and Go trials at Cz, shown for the analysed frequencies (1 to 30 Hz) and separately for 50% (left side) and 72% go blocks (right side) and for the easy (upper half) and hard condition (lower half). The white square marks the analyzed time and frequency range for the nogo-related theta band increase with the respective topographic map. **B **Group differences in the time frequency data. Depicted is the power change relative to the baseline between Nogo and Go trials at Cz, separately for the two groups (4rep: upper part; 7rep: lower part) and 50% (left) vs. 72% go blocks (right side). The white squares indicate the analyzed time and frequency range for the beta and theta band, and shown are the topographies for the beta [scale -20 to 20% power change; collapsed across 50 and 72% blocks] and theta band changes [scale -40 to 40% power change] in the 4rep and 7rep group.

**Figure 4:**
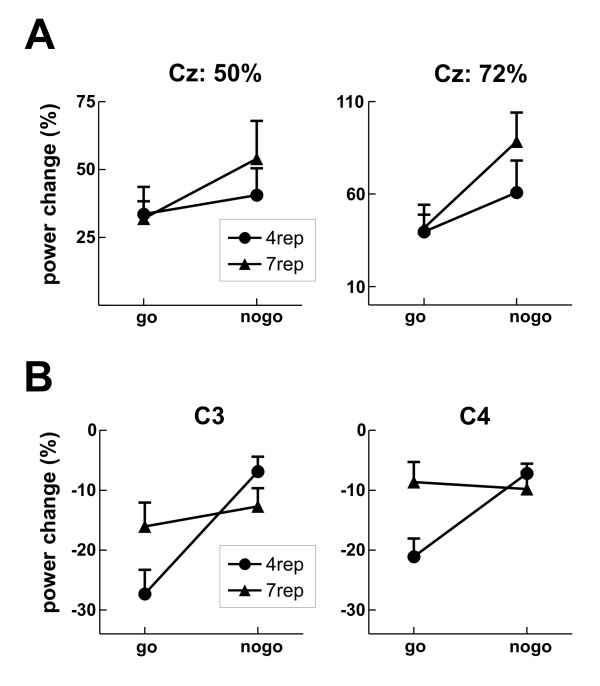
**Group differences in the time frequency results**. **A **Results for the theta band response (4 - 8 Hz, 300 - 500 ms) with the average power change relative to the baseline for both groups in go and nogo trials at Cz, separately for 50% (left side) and 72% go blocks (right side). **B **Results for the beta band response (20 - 30 Hz, 400 - 600 ms). Shown is the average power change relative to the baseline for both groups in go and nogo trials, separately for the left hemisphere (electrode C3, left side) and right hemisphere (electrode C4, right side).

Regarding the time-frequency domain, we observed an increase of power in the theta band (4-8 Hz) in nogo compared to go trials between 300 - 500 ms, which was most prominent at central electrodes, and a decreased beta band response in go compared to nogo-trials between 400 - 600 ms (Figure 3A and 4B). Moreover, first visual inspection suggested an enhanced theta band response in the 7rep compared to the 4rep group, together with a stronger beta band decrease in the 4rep group (Figure 3B and 4B). Effects of the conditions and group differences will be reported first for the theta-band and then for the beta band.

#### Theta response

To investigate the theta effect, we subjected the mean power increase between 300 - 500 ms and 4 - 8 Hz to a repeated measures ANOVA with the between-subject factor DRD4 and the within-subject factors Go (go vs. nogo), Discriminability (easy vs. hard), Probability (50% vs. 72% go) and Electrode (Fz, Cz, Pz). A significant interaction of Go*Probability*Electrode (F_2,36 _= 7.02, p = 0.003) reflected the enhanced theta band response in nogo vs. go trials, which was found to be increased in the 72% go compared to the 50% go block (interaction Go*Electrode in 50% block: F_2,36 _= 8.23, p = 0.001; in 72% block: F_2,36 _= 16.37, p < 0.001). In both, the 50% and 72% go blocks, the theta increase was most pronounced at Cz, which can be observed in the topographic map in Figure 3A.

Importantly, we observed an interaction of this effect with the group factor (DRD4*Go*Probability*Electrode: F_2,36 _= 8.52, p = 0.001, partial η^2 ^= 0.321; DRD4*Go: F_1,18 _= 3.12, p = 0.094), such that the nogo-related increase in the theta band was significantly higher in the 7rep (Go*Probability*Electrode: F_2,18 _= 10.72, p = 0.001) compared to the 4rep group (Go*Probability*Electrode: F_2,18 _= 4.55, p = 0.025). The theta increase was in both groups higher in 72% compared to 50% blocks and was most pronounced at Cz (Figures 3B and 4A). In fact, at the single electrode level, we observed in the 4rep group no significant main effects of Go in the 50% block (all electrodes: p > 0.05) and only at Cz for the 72% block (p < 0.01), whereas the 7rep group showed significant effects at Cz for the 50% block (p < 0.05) and at all midline electrodes for the 72% block (Fz and Cz: p < 0.01, Pz: p < 0.05).

#### Beta response

In addition to the theta band response, we observed differences between go- and nogo-trials in the beta band (maximum at 20 - 30 Hz) between 400 to 600 ms (Figure 3B). More specifically, go-trials led to a more pronounced power decrease compared to nogo-trials (main effect Go: F_1,18 _= 6.44, p = 0.021), which was found to be more pronounced over the left than right hemisphere (Go*Hemisphere: F_1,18 _= 9.14, p = 0.007). Interestingly, this go-related desynchronization was observable in the 4rep group only (DRD4*Go: F_1,18 _= 10.45, p = 0.005, partial η^2 ^= 0.37; 4rep group: Go: p < 0.001), whereas no differences between go- and nogo-trials were seen in the 7rep group (Go: F < 1) (Figure 3B and 4B).

## Discussion

The present study aimed at investigating the role of the DRD4 VNTR polymorphism in the modulation of prefrontal processes related to response inhibition. To this end, we compared homozygous 4rep and 7rep carriers with respect to neurophysiological markers of response inhibition. Carriers of the 7rep allele, which is suspected to bear a higher risk for ADHD, performed more accurately in the Go/Nogo-task than carriers of the 4rep allele, but the difference yielded marginal significance only. The same group showed an enhanced nogo-related theta band increase and an absent go-related beta band decrease, whereas no genetic effects on ERPs could be detected.

### Behavior

Behaviorally, 7rep carriers showed more accurate performance in the task, whereas the 4rep carriers performed less accurately (less hits) in particular in the more demanding condition (72% go blocks). Our results are in partial contrast to recent results of Congdon and co-workers [30], who reported *impaired *response inhibition in a stop-signal task in carriers of at least one 7rep allele. However, this effect was particularly observable in combination with a dopamine transporter polymorphism (DAT1), and the authors included both heterozygous and homozygous participants for the DRD4 polymorphism, which makes a direct comparison of the results difficult. It is notable also that a reduced hit rate can hardly be explained with differences in inhibitory control but rather with differences in sustained attention between the groups. Differences in accuracy are also likely to result from differences in cortical activity which fits well with the assumed role of DRD4 in cortical, especially prefrontal areas [14,15]. In line with neuropsychological studies in ADHD patients with the 7rep allele [2,7,8], our results rather support the view that this variant is associated with unimpaired or in our case even improved cognitive control functions.

### Event-related potentials

Unexpectedly, we did not find any group differences in the inhibition-related ERPs, namely the nogo-N2 and P3. Both components are frontocentrally distributed and are observed in experimental settings calling for an inhibition of motor responses as in Go/Nogo tasks [36,37]. The N2 is suggested to reflect inhibitory mechanisms emanating from areas in the prefrontal cortex [11,53]. This view has been challenged, though, by others who see the N2 in go/nogo tasks as an index of conflict monitoring [38]. Interestingly, both accounts (inhibition and conflict monitoring) of the N2 predict and have previously shown an enhanced N2 in blocks of high go-probability, which we did not observe in the present data [38,54]. As noted in the results section, there was a tendency for an interaction between discriminability and go-probability on the N2, which has not been addressed in previous work. The probability effect on reaction times was also stronger for the easy compared to the hard condition. As this is to our knowledge the first study to look at this interaction of go-probability and stimulus discriminability, future studies will need to replicate this finding before drawing conclusions with respect to the inhibition and conflict-monitoring accounts of the nogo-N2.

The nogo-P3 on the other hand has rather been related to a later stage of the inhibitory process, indexing the monitoring of its successful implementation instigated by the anterior cingulate cortex or pre-SMA [12]. Given previous reports of altered nogo-related N2/P3 components in ADHD [11,12] and the outlined expression profile of the D4 receptor [15], one might expect a modulation of these prefrontal components by the DRD4 polymorphism. However, the N2 was in our case largely overlapping with the posterior P3b effect, an enhanced positivity for Go- compared to Nogo-trials. As has been pointed out in the results section, this overlap makes a clear assessment of group differences in the nogo-N2/P3 difficult.

### Time-frequency data

We additionally performed time-frequency analyses of the data, which have been demonstrated to be a useful tool to decompose underlying functional components of ERPs [55,56]. Moreover, they enable us to study oscillations in higher frequency bands (beta band), which are of lower amplitude and possibly less phase-locked to the trigger event and therefore less detectable in ERPs. We observed an increase in the theta band in nogo- compared to go-trials, which was additionally enhanced in 72% go blocks. These results are in line with previous findings of an inhibition-related increase in the theta band [40], which is probably stemming from activity in the PFC and/or ACC [43-45]. Moreover, modulations of oscillations in the theta band have been consistently found in tasks calling for increased cognitive effort and action control as in interference resolution, error-related processing or working memory [44,45,57,58]. Interestingly, 7rep participants showed a significantly higher nogo-related theta band response compared to the 4rep group. Both groups showed the maximum over central electrodes and a stronger theta band response in 72% compared to 50% go blocks. This points to an elevated level of inhibition-related prefrontal activity in the 7rep participants.

Moreover, we detected group differences in the beta band response, such that only 4rep carriers presented the typical go-related beta decrease, whereas no go-related beta decrease was observable in the 7rep group. Oscillations in the beta band have been linked to motor responses with a typical decrease during the motor response and a subsequent increase (event-related desynchronization, ERD, and synchronization, ERS, respectively), stemming from the sensorimotor and primary motor areas [46]. A reduced ERD is typically seen in relation to inhibition of the motor response [42,47]. As the stimulus onset asynchrony in the present study was fixed to only 1000 ms, our design did not allow to analyze ERD/ERS directly, because a longer intertrial interval is needed to have a reliable baseline [46]. A reduced beta decrease has been found also in relation to post-error-slowing which was taken as evidence for inhibitory activity underlying post-error adaptation [41]. Notably however, the post-error related beta effect had a more frontal topography than the current beta response in the 4rep group. Importantly, genetic effects in the present study were seen in the go-related beta decrease but not in the nogo-related beta response. The lack of a go-related decrease in the beta band in the 7rep group suggests that the enhanced prefrontal activity led to a generally stronger inhibitory bias in these participants.

At first glance, enhanced prefrontal functions and better response inhibition seem to be at odds with the suggested increased risk for ADHD development. ADHD children have been shown to perform worse in cognitive control and response inhibition tasks, reflected especially in reduced accuracy and increased reaction time variability rather then a general reaction time decrement [49,59]. They moreover present a reduced level of inhibition-related prefrontal activity in fMRI and ERP studies [11-13,49,59]. However, as outlined in the introduction, some behavioral studies in ADHD children reported abnormal behavior in children with the 7rep variant, but unimpaired cognitive functions, despite the allele's robust link with the disease [2,7]. These data converge on a differential role of this polymorphism in terms of cognitive functions and of ADHD related behavior. Interestingly, studies in rodents pointed to positive effects of prefrontal D4 receptor blockade on different cognitive functions, possibly because of the receptor's inhibitory effect on glutamatergic activity [60,61]. Floresco and co-authors [61], for instance, reported improved behavior in a maze-based set-shifting task after infusion of a D4 receptor antagonist in the PFC and impaired performance after infusions of a D4 receptor agonist. Given the observed reduced dopaminergic response in the 7rep variant [26], these results hint at a possible mechanism to explain the polymorphism's effect on prefrontal functions. Further studies will be needed to delineate the polymorphism's specific effects on cognitive functions.

Obviously the present results have to be interpreted with caution because of rather small sample size due to the scarcity of 7rep homozygotes. Replications of these results in independent samples are clearly needed. It should be noted also that the effects both on the behavioral and electrophysiological level were surprisingly small given the quite robust association findings of this polymorphism with ADHD. Importantly, in contrast to previous studies on the DRD4 VNTR, we included homozygous participants only, which allows clear interpretations of the results, whereas the physiological effects in heterozygotes are difficult to judge for this gene.

## Conclusions

The present study is the first to investigate the neurophysiological basis of the DRD4 VNTR association with ADHD by examining its effects on neural correlates of inhibitory functions in a healthy sample. Genetic effects on the levels of behavior and neurophysiology provide further evidence for the polymorphism's role in the modulation of prefrontal functions and dovetail previous findings on the impact of dopaminergic genes on cognitive control functions [20,50]. The results underline behavioral findings of improved cognitive functions in 7rep carriers, which is contrary to the allele's association with ADHD. We suggest on the basis of the present findings that the 7rep variant might entail better cognitive control due to the D4 receptor's modulatory function in prefrontal areas. Neurophysiological studies (EEG or fMRI) might be thus a more promising research line to investigate the role of certain genes in the development of ADHD, going beyond mere association or behavioral studies [62].

## Methods

### Participants

The genotyping was performed in a large sample of 656 students from the University of Barcelona (491 women; age range from 18 to 56, mean = 21.7, S.D. = 3.5), who underwent a comprehensive neuropsychological test battery and filled out several personality questionnaires. We initially performed the genotyping for six different polymorphisms in the dopaminergic system, namely COMT Val108/158Met, DRD4 -521, DRD4 120bp, DRD4 exon III, MAO-A 30bp and DAT1 VNTR. In the present study, we focused on the DRD4 exon III polymorphism only. The allele frequencies for this polymorphism were as follows: 8.7% (2 repeat), 2.7% (3 repeat), 70.9 (4 repeat), 1.5% (5 repeat), 0.3% (6 repeat) and 15.9% (7 repeat). The observed genotype frequencies were in Hardy-Weinberg equilibrium (chi^2 ^(27) = .2369, n.s.). 29 participants were homozygous for the 7 repeat allele. Note that the selection of this polymorphism was based on previous literature only and not on behavioral results of the neuropsychological test battery. The EEG paradigm (see below) was performed in the sub-sample only and we did not test for any effects of other polymorphisms in this sample. Previous EEG studies with other sub-samples of this large group and related to other polymorphisms have been published elsewhere [20,63].

We selected 27 (22 women; age range from 20 to 30 years, mean = 23.0) participants based on their DRD4 exon III alleles for the second session. Of these participants, 11 (9 women) were homozygous for the 7 repeat version (in the following referred to as 7rep group) and 16 (13 women) were carriers of two 4 repeat alleles (in the following referred to as 4rep group). One of the 7rep and four of the 4rep participants had to be excluded because of extensive artefacts in the EEG data. One of the participants (4rep group) could not discriminate between the go and nogo stimuli in the hard condition (see below for explanation of the paradigm) leading to a high amount of false alarms (70% in the 50% go block and 44% in the 72% go block). One participant (4rep group) reported to have had epileptic seizures in the past. We included thus 20 subjects in our final analyses, 10 in each group. They were all right-handed participants of European ancestry (except one from Ecuador) and were free of neurological and psychiatric disorders (self-report). They were paid for their participation and gave written informed consent. All procedures were approved by the local ethical Institutional Review Board (IRB00003099).

### Genotyping

DNA contributed to the study was prepared by standard techniques from two independent EDTA blood samples of each participant. DNA was amplified with fluorescent primers: DRD4_ExIII_for: 5'-Fam-GCGACTACGTGGTCTACTCG-3', DRD4_ExIII_rev: 5'-AGGACCCTCATGGCCTTG-3'. 2mM MgCl2 and 10% Q-Solution were added to the reaction mix. Due to reduced amplification of the 7 repeat allele in comparison to shorter repeat alleles, the elongation time was increased. The following cycling conditions were used: 95°C 15', (98°C 15'', 62°C 30'', 72°C 1') × 34 cycles, 72°C 10'. To determine fragment length PCR products were analyzed on an ABI 3100 automated sequencer with a fluorescence detection system.

Genotypes of participants selected for ERP were controlled in an independent second DNA sample by direct sequencing using the ABI PRISM BigDye Terminator v3.1 Cycle Sequencing Kit (Applied Biosystems, Foster City, USA). Sequencing products were resolved on an ABI 3100 automated sequencer (Applied Biosystems, Foster) and analyzed using the Staden Package [64].

### Go/Nogo-Paradigm

We adapted a hybrid choice-reaction go/nogo task introduced by Osman and colleagues [65]. In this task, two letter-digit pairs serve as stimuli, with one pair being easily discriminable (V and 5) and another pair being hard to discriminate ([letter] l and [number] 1). One stimulus at a time was presented on the left or right side of a fixation cross, asking for left or right hand responses, respectively. The two response hands and the two conditions (easy vs. hard) were equally frequent and randomly presented within each block of the experiment. Half of the participants were instructed to respond to letters in the first part of the experiment and to digits in the second part of the experiment, and vice versa for the other half of the participants. We additionally manipulated the probability of go trials, including blocks with either 50% go trials (1^st ^half) or 72% go trials (2^nd ^half). Again, the order of blocks with 50% or 72% go probabilities was counterbalanced across participants. This task thus enabled us to parametrically manipulate inhibitory functions: The hard condition and blocks with a higher go probability were expected to put higher demands on inhibitory control and thereby increase nogo-related neurophysiological responses.

The stimuli were presented for 50 ms and the stimulus onset asynchrony was fixed to 1000 ms. Total number of trials was 912 in the 50% go condition and 1000 in the 72% go condition. The experiment began with practice trials to familiarize participants with the task. They were instructed to respond as fast and accurate as possible. After every 25 trials, a short break of 7 seconds was included to allow participants to blink and about every 2 minutes a longer break was included. The total duration of the experiment was about one hour.

### ERPs

The electroencephalogramm (EEG) was recorded from 29 tin electrodes mounted in an elastic cap (electrode positions: Fp1/2, F3/4, C3/4, P3/4, O1/2, F7/8, T3/4, T5/6, FC1/2, FC5/6, CP1/2, CP5/6, PO1/2, Fz, Cz, Pz) with reference electrodes placed on the right and left mastoids. During recording, all scalp electrodes were referenced against the right mastoid and offline re-referenced against the algebraic mean of the activity at the two mastoid electrodes. Electrode impedances were kept below 5 kΩ. Horizontal eye movements and blinks were monitored by an electrode placed on the outer canthus of the right eye and below the right eye. EEG and EOG were recorded continuously and digitized with a sampling rate of 250 Hz (bandpass from 0.01 to 70 Hz). After rejection of eye and muscle artefacts using rejection criteria set individually after determining the typical amplitude of the respective artefacts in the given individual, stimulus-locked averages were obtained for the different conditions (-100 to 924 ms) with the 100 ms preceding the stimulus considered as baseline.

For statistical analyses, mean amplitudes were subjected to a repeated measures ANOVA with the between-subject factor DRD4 (4rep vs. 7rep) and the within-subject factors Go (Go vs. Nogo), Discriminability (Easy vs. Hard), Probability (50% vs. 72% go trials) and electrode position (as stated below). As we were interested in the nogo-related N2 and P3, we considered relevant only main effects or interactions involving the factor Go. We refer to the frontal P3, which is higher in nogo compared to go trials as "nogo-P3" [37] to dissociate it from the posterior P3b, which is higher for go compared to nogo-trials. The analyzed time windows were chosen in light on previous results. For all statistical effects involving more than one degree of freedom in the numerator, the Huynh-Feldt correction was applied to correct for possible violations of the sphericity assumption [66] and the corrected probabilities are reported.

### Time-frequency analyses

To study the inhibition-related oscillatory activity, single trial data were convolved with a complex Morlet wavelet:

with the relation *f*_0_/*σ*_*f *_(where *σ*_*f *_= 1/(2*πσ*_*t*_)) set to 6.7 [67]. For single trials in the different conditions, we computed and averaged for each subject changes in time varying energy (square of the convolution between wavelet and signal) in the studied frequencies (from 1 to 30 Hz; linear increase) with respect to baseline. Based on previous studies, we were interested in changes in the theta band (4 - 8 Hz) reflecting prefrontal control functions [40,44,58] and the beta band, which is rather related to the motor output itself [46]. Selection of the analyzed beta frequencies (20 - 30 Hz) was based on visual inspection of the main go-related effect. The selection of the analyzed time windows was based on the visual inspection of the maxima in the theta and beta band response. Mean increase/decrease in power was obtained for the three midline electrode locations (Fz, Cz, Pz) and entered into a repeated measures ANOVA with the within-subject factors Go (Go vs. Nogo), Discriminability (Easy vs. Hard), Probability (50% vs. 72% go trials) and Electrode (Fz, Cz, Pz) and the between-subject factor DRD4 (4rep vs. 7rep). As first visual inspection suggested a lateralized effect in the beta band, we analyzed the beta response at the electrodes F3/4, C3/4 and P3/4 yielding a repeated measures ANOVA with the factors DRD4, Go, Probability, Discriminability, Anterior-Posterior (three levels: frontal, central, posterior) and Hemisphere (right vs. left). As for the ERPs, we were interested only in main effects or interactions of the factor Go. For all interactions with the factor Group, partial eta square (partial η^2) ^are given as measure of the effect size.

## Authors' contributions

UMK performed the electrophysiological study, analyzed the data, wrote the manuscript; NR performed the electrophysiological study; RS performed the genetic analyses; TC performed the behavioural testing of the original sample; LS co-designed study and performed genetic analysis; JMP contributed to the analysis and behavioural testing; DC performed the behavioural testing of the original sample; EC contributed to the analysis; ARF co-designed the study, contributed to the manuscript; TFM co-designed the study, contributed to the manuscript.
